# Identification of novel biomarkers involved in oral squamous cell carcinoma by whole transcriptome sequencing and bioinformatics analysis

**DOI:** 10.1186/s12935-025-03913-9

**Published:** 2025-07-22

**Authors:** Hongliang Du, Zhenze Wang, Mengyi Qi, Yunqing Pang, Qingling Lin, Dengqi He, Jing Wang

**Affiliations:** 1 The First Clinical Medical College of Lanzhou University, Lanzhou, 730000 China; 2 Department of Oral and Maxillofacial Surgery, The First Hospital of Lanzhou University, Lanzhou, 730000 China; 3 Department of Stomatology, The First Hospital of Lanzhou University, Lanzhou, 730000 China; 4 School of Stomatology, Lanzhou University, Lanzhou, 730000 China; 5 Department of Stomatology, The First People’s Hospital of Lanzhou City, Lanzhou, 730000 China; 6 Department of Intensive Care Unit, The First Hospital of Lanzhou University, Lanzhou, 730000 China; 7 Clinical Research Center for Oral Diseases, Lanzhou, 730000 China

**Keywords:** Oral squamous carcinoma, Complete RNA sequencing, Molecular indicators, Regulatory networks, Drugs

## Abstract

**Background:**

Oral squamous cell carcinoma (OSCC) is among the most common malignant tumors in the oral and maxillofacial regions, characterized by high drug resistance and poor treatment outcomes. This underscores the urgent need to identify novel biomarkers for OSCC.

**Methods:**

Differentially expressed messenger RNAs (mRNAs), microRNAs (miRNAs), and long non-coding RNAs (lncRNAs) (DE-mRNAs, DE-miRNAs, and DE-lncRNAs) between primary and control groups, as well as metastatic and primary groups, were identified using whole transcriptome sequencing data. Candidate OSCC genes were derived from DE-mRNAs. Potential biomarkers were then identified using five algorithms from CytoHubba. Biomarkers were validated via univariate Cox regression and Kaplan–Meier (K–M) survival analysis. Additional analyses included subcellular localization, mutation analysis, and Gene Set Enrichment Analysis (GSEA). Key drugs for OSCC treatment were also identified. Quantitative real time polymerase chain reaction (qRT-PCR) and immunohistochemistry were employed to verify the expression levels of key biomarkers.

**Results:**

A total of 304 candidate genes were identified, with 29 potential biomarkers selected by five algorithms. ANPEP, APOB, GLP1R, and SI exhibited significant survival differences in the K–M curves, establishing them as OSCC biomarkers. These biomarkers were predominantly localized in the cytoplasm, with SI and APOB showing the highest mutation susceptibility. Enrichment analysis revealed that the ‘interferon-gamma response’biological function was co-enriched by ANPEP, APOB, and SI. Furthermore, BIBW2992 (afatinib) and PF.02341066 (crizotinib) were most strongly correlated with the biomarkers, suggesting their potential as key drugs for OSCC treatment. Additionally, the findings were validated by qRT-PCR and immunohistochemical analyses, and the results were consistent with the RNA-seq data.

**Conclusion:**

ANPEP, APOB, GLP1R, and SI were identified as potential OSCC biomarkers, offering valuable insights for further research and therapeutic development.

**Supplementary Information:**

The online version contains supplementary material available at 10.1186/s12935-025-03913-9.

## Introduction

Oral squamous cell carcinoma (OSCC) is the most prevalent head and neck malignancy, representing a significant threat to global health with approximately 377,713 new cases and 177,757 annual deaths worldwide [1]. Due to challenges in early detection and a lack of effective treatments, the 5-year survival rate remains only 30% in advanced stages [2]. Patients with metastatic or late-stage OSCC face poor prognoses and diminished health-related quality of life [3, 4]. Treatment typically involves a combination of surgery, radiation, and chemotherapy [5]; however, the efficacy is limited, as most patients are diagnosed at advanced or metastatic stages.

With the rapid development of precision medicine, the importance of biomarkers in guiding treatment choices for OSCC is becoming increasingly prominent, making them a key tool for personalized therapy. For instance, Shin Tojo et al. utilized spatial transcriptomics technology and discovered that CXCL13 is specifically overexpressed in primary OSCC tissues. This finding has the potential to become a novel biomarker for OSCC, providing a new direction for clinical diagnosis and therapeutic strategies [6]. Additionally, Soni Shaikh et al. revealed changes in key metabolites in oral cancer through proton nuclear magnetic resonance spectroscopy, identifying metabolic biomarkers such as CD39 and CD73 that have potential application value in the diagnosis and prognostic assessment of OSCC [7]. These studies indicate that biomarkers play a significant role not only in early diagnosis but also in accurately assessing patient prognosis, providing a more reliable theoretical basis for personalized treatment. Therefore, the identification of novel biomarkers is crucial for improving early diagnosis and facilitating the development of innovative therapies, alongside a deeper understanding of OSCC's pathogenesis and molecular mechanisms.

In the past decade, whole transcriptome sequencing has become an essential tool for elucidating the genetic mechanisms underlying various cancers [8, 9]. Tumor-specific mutations and recurrent inter-chromosomal fusion genes have been identified in OSCC through this technology [10, 11]. Despite these advancements, the molecular mechanisms underlying metastasis in OSCC remain poorly understood. Whole transcriptome sequencing offers a promising approach to investigating the roles of messenger RNAs (mRNAs), microRNAs (miRNAs), and long non-coding RNAs (lncRNAs) in OSCC and their potential involvement in metastasis.

The rapid advancement of high-throughput sequencing technologies has led to significant findings regarding the role of non-coding RNAs (ncRNAs) in various biological processes, including gene regulation, cellular processes, and epigenetic modifications. NcRNAs, such as small interfering RNAs (siRNAs), miRNAs, and lncRNAs, are not translated into proteins but play critical roles in regulating cellular functions. Studies have shown that ncRNAs contribute to OSCC drug resistance through multiple mechanisms, including modulation of transmembrane transport proteins, epithelial-mesenchymal transition (EMT), DNA damage repair, and autophagy [12, 13]. Furthermore, ncRNAs are closely linked to the growth and survival of OSCC cells by disrupting the balance between proliferative signaling and growth suppression [14]. Despite these findings, the precise mechanisms by which ncRNAs influence OSCC remain insufficiently explored, warranting further investigation.

This study aims to identify novel biomarkers involved in OSCC metastasis. Whole transcriptome sequencing was performed on adjacent cancer tissues, primary OSCC samples, and metastatic OSCC samples, with differentially expressed mRNAs, miRNAs, and lncRNAs identified. Through bioinformatics analysis, the study explores the biological functions, signaling pathways, mutation profiles, and drug sensitivity of OSCC. The findings are expected to significantly enhance the understanding of OSCC pathogenesis, provide new insights into prognostic prediction, and offer potential therapeutic applications for mRNAs, miRNAs, and lncRNAs in OSCC metastasis.

## Materials and methods

### Associated data

Transcriptome sequencing data from tissues of five patients with metastatic OSCC (metastatic group), five patients with primary OSCC (primary group), and five peri-tumor tissues (control group) were used as the training set for this study. The TCGA-OSCC dataset was retrieved from The Cancer Genome Atlas (TCGA, http://cancergenome.nih.gov/), comprising transcriptome sequencing data from the oral cavity, floor of the mouth, jaw, and buccal mucosa. The sample collection method of this study was prospective, and all samples were obtained from the First Hospital of Lanzhou University. The study was reviewed and approved by the hospital's ethics committee (Ethics approval number: LDYYLL2024-776), adhering to relevant ethical guidelines to ensure that patients provided informed consent and their privacy was protected. Additionally, this study selected matched samples of primary, metastatic tumors, and normal tissues from the same patient. To ensure the integrity of RNA for subsequent experiments, the samples were processed with formalin-fixed paraffin-embedded (FFPE) treatment. Specific clinical information of the pathological samples is presented in Supplementary Table 1 according to the 8th edition of the TNM classification for head and neck tumors [15]. Control samples were taken from the tissue surrounding the tumor, at least 1 cm away from the tumor edge, to ensure no histological signs of atypical hyperplasia. The TCGA-OSCC dataset, which includes survival information, contains 362 samples, consisting of 330 disease samples and 32 control samples. This dataset was used to evaluate the prognostic value of identified biomarkers.The specific information of the dataset samples was as follows: this dataset was used to evaluate the prognostic value of identified biomarkers. The disease samples were tested using In Situ Hybridization (ISH) as the detection method, resulting in 4 Human Papillomavirus (HPV) Positive, 43 HPV Negative, and 283 HPV Not Available cases. When tested using p16INK4a (p16) as the detection method, there were 8 HPV Positive, 51 HPV Negative, and 271 HPV Not Available cases.The margin status of the control samples was categorized into four types: 4 cases were classified as Close, 22 cases as Negative, 4 cases as Positive, and 2 cases as Not Available.

### Whole transcriptome sequencing

#### The miRNA library was built for sequencing

Total RNA was extracted from samples using the TRIzol Total RNA Extraction Kit (Invitrogen, CA, USA). After extraction, the purity and integrity of the RNA were detected by instruments such as 1% agarose gel electrophoresis, NanoDrop spectrophotometer (Thermo Scientific, DE, USA), Qubit^®^ 2.0 Flurometer (Life Technologies, CA, USA), and Bioanalyzer 2100 system (Agilent Technologies, CA, USA). After the samples passed the quality inspection, 3 μg of RNA was taken to construct a library using the Small RNA Sample Pre Kit. Utilizing the special structures at the 3′ and 5′ ends of Small RNA (a complete phosphate group at the 5' end and a hydroxyl group at the 3′ end), with total RNA as the starting sample, adapters were directly added to both ends of Small RNA, and then cDNA was synthesized by reverse transcription. After the library construction was completed, the insert size of the library was detected using Agilent 2100 to ensure that it met the requirements of the sequencing platform, thereby guaranteeing the accuracy and reliability of the sequencing data. After the insert size was in line with expectations, single-end 50 bp (SE 50) sequencing was carried out on the Illumina Novaseq 6000 platform.

#### The lncRNA library was built for sequencing

For mRNA extraction, 3 µg of RNA was processed with the Epicentre Ribo-zeroTM ribosomal RNA (rRNA) Removal Kit (Epicentre, USA), eliminating rRNA to obtain mRNA. A strand-specific library was then constructed using the NEBNext^®^ UltraTM Directional RNA Library Prep Kit for Illumina^®^ (NEB, USA). After quality control, the library was sequenced on the Illumina Novaseq 6000 PE150 sequencing platform to obtain miRNA-related data.

### Analysis of miRNA, mRNA, and lncRNA data

The adapters, duplicate sequences, and low-quality sequences were filtered using ‘cutadapt’ (version 1.18) and ‘Trimmomatic’ software (version 0.33) [16], resulting in high-quality clean reads. Cleaned mRNA-seq and lncRNA-seq data were aligned to the reference human genome (GRCh38.p13) using ‘hisat2’ (version 2.2.1), while miRNA-seq data were mapped to human miRNA annotations from miRBase (http://www.mirbase.org/) using ‘Bowtie’.

### Differential analysis of biological pathways

To evaluate the reproducibility among samples and ensure data quality, the principal component analysis (PCA) was first carried out on the whole transcriptome sequencing data. Following data quality assessment, gene set variation analysis (GSVA) was conducted on the control, primary, and metastatic groups using the ‘GSVA’ package (version 1.49.4) with the ‘HALLMARK’ gene set. Biological pathways that exhibited significant differences between the groups were selected.

### Identification of differentially expressed mRNAs, miRNAs, and lncRNAs (DE-mRNAs, DE-miRNAs, and DE-lncRNAs) between groups

DE-mRNAs, DE-miRNAs, and DE-lncRNAs between the primary and control groups, as well as metastatic and primary groups, were identified using ‘DESeq2’ (version 1.26.0) with criteria of *p* < 0.05 and |log_2_ fold change (FC)|> 1 [17].

### Acquisition of candidate genes and their enrichment analysis

To identify candidate genes for OSCC, the portion of DE-mRNA2 (metastatic and primary groups) shared with DE-mRNA1 (primary and control groups) was removed, and the remaining genes were recorded as candidates. The aim was to explore genes with unique expression changes during the transition of a tumor from a primary to a metastatic state. The same approach was applied to DE-miRNAs and DE-lncRNAs to identify candidate miRNAs and lncRNAs, respectively. Enrichment analysis of candidate genes was performed using the ‘clusterProfiler’ package (version 3.14.3) to explore Gene Ontology (GO) and Kyoto Encyclopedia of Genes and Genomes (KEGG) pathways (*p* < 0.05).

### Construction of regulatory networks

To investigate the molecular regulatory mechanisms of the candidate genes, upstream miRNAs were predicted using the starBase database (http://starbase.sysu.edu.cn/), and intersections with the candidate miRNAs were taken to identify target miRNAs. Subsequently, the upstream lncRNAs were predicted based on the target miRNAs in the starBase database, and intersections with candidate lncRNAs were identified as target lncRNAs. A competing endogenous RNA (ceRNA) regulatory network was then constructed using ‘Cytoscape’ (version 3.9.0). Additionally, protein–protein interaction (PPI) networks (confidence > 0.7) of the candidate genes were constructed using STRING (https://string-db.org/).

### Identification of biomarkers

Based on the constructed PPI network, the top 50 candidate genes for each of the five algorithms in CytoHubba (Degree, Maximal Clique Centrality (MCC), Closeness, Radiality, and Stress) were selected based on their calculated scores. The intersection of these top genes was recorded as potential biomarkers for OSCC. Univariate Cox regression analysis was performed on patients with survival data from TCGA-OSCC using the ‘survival’ package (version 3.5–3) to screen for prognosis-related genes (*p* < 0.05). The patients were then divided into two groups (high and low expression) based on the optimal expression values of each biomarker. Kaplan–Meier (K–M) survival curves were plotted using the ‘survminer’ package (version 0.4.9), and genes showing significant differences in survival between groups were identified as OSCC biomarkers.

### Association analysis between biomarkers and clinical features

To assess the relationship between biomarkers and clinical features (age, gender, pathologic T stage, pathologic N stage, overall stage, and overall survival), TCGA-OSCC patients were stratified into subgroups based on these features. The Wilcoxon test (*p* < 0.05) was used to compare biomarker expression differences between subgroups.

### Subcellular localization and mutation analysis of biomarkers

Sequences of the biomarkers were retrieved from the National Center for Biotechnology Information (NCBI, https://www.ncbi.nlm.nih.gov/), and the mRNA Locater database (http://bio-bigdata.cn/mRNALocater) was used to predict the subcellular localization of the biomarkers. Additionally, mutations in the biomarkers in TCGA-OSCC samples were analyzed using the ‘maftools’ package (version 2.8.0) in combination with the TCGA database.

### Functional enrichment analysis of biomarkers

The correlation coefficients for genes across all samples in the metastatic and primary groups were calculated and ranked in descending order. These genes were then subjected to gene set enrichment analysis (GSEA) using the ‘clusterProfiler’ package to explore the potential functions of the biomarkers (*p* < 0.05), with the ‘HALLMARK’ gene set used as the reference.

### Analysis of immunological features and drug susceptibility analysis

Immune profiles associated with OSCC were retrieved from the Tumor and Immune System Interaction Database (TISIDB, http://cis.hku.hk/TISIDB/index.php). Spearman's correlation analysis was performed to assess the relationships between biomarkers and various immune profiles in the metastatic and primary groups. To investigate the relationship between biomarkers and drug sensitivity, targeted therapeutic agents for OSCC were obtained from the Genomics of Drug Sensitivity in Cancer (GDSC, https://www.cancerrxgene.org/). The 50% inhibitory concentration (IC_50_) values for drugs acting on OSCC were determined for TCGA-OSCC samples using the ‘pRRophetic’ package (version 0.5). Correlations between biomarkers and IC_50_ were evaluated using Spearman’s correlation analysis, and drugs with correlation coefficients greater than 0.4 were selected as key therapeutic agents.

### Construction of transcription factor (TF)-biomarker regulatory network and biomarker-disease co-expression network

TFs associated with biomarkers were predicted using the JASPAR database (https://jaspar.genereg.net), and a TF-biomarker regulatory network was constructed. Additionally, diseases significantly associated with the biomarkers were identified through the Disease Gene Network database (DisGeNet, http://www.disgenet.org), and a biomarker-disease co-expression network was built using Network Analyst. Furthermore, biomarker expression was examined in TCGA-OSCC samples to compare OSCC and control groups, and the expression levels were validated using sequencing data from control, primary, and metastatic groups.

### Quantitative real time polymerase chain reaction (qRT-PCR) and Immunohistochemistry validation of the key biomarkers

Total RNA was extracted from tissues (from patients) using TRIzol reagent(Takara), according to the manufacturer's protocols. We obtained cDNA using the PrimeScriptTM RT reagent Kit with gDNA Eraser(Takara). QRT-PCR was performed using the Takara SYBR Premix Ex TaqII with an ABI 7500 system. The primers used for qRT-PCR were as follows: ANPEP Forward: 5′-GACCAAAGTAAAGCGTGGAATCG-3′, Reverse: 5′-TCTCAGCGTCACCCGGTAG-3′; APOB Forward: 5′-ACACACTGGACGCTAAGAGGA-3′, Reverse: 5′-ACTTGTGCTACCATCCCATACT-3′ GLP1R Forward: 5′-GGTGCAGAAATGGCGAGAATA-3′, Reverse: 5′-CCGGTTGCAGAACAAGTCTGT-3′, and SI Forward: 5′-TCCAGCTACTACTCGTGTGAC-3′, Reverse: 5′-CCCTCTGTTGGGAATTGTTCTG-3′. The relative mRNA expression was calculated as 2^−ΔΔCt^. β-actin mRNA were used as normalization standards.

Histopathological examination of tissues was performed using hematoxylin and eosin (H&E) staining and immunohistochemical (IHC) analysis. For H&E staining, tissue samples were fixed in 4% paraformaldehyde solution for 30 min, embedded in paraffin, and sectioned into 5-µm-thick slices. Standard histological staining techniques were applied to assess the differentiation degree of tumor cells and to evaluate the metastatic status in lymph nodes. For IHC analysis, paraffin-embedded tissue sections were deparaffinized using xylene, rehydrated through a graded ethanol series, and subjected to antigen retrieval with ethylenediaminetetraacetic acid (EDTA) at pH 8.0 for 15 min in a pressure cooker. IHC staining was performed using primary antibody overnight at 4 ℃. The primary antibodies used were: ANPEP Rabbit pAb (1:100; APR25992N), APOB antibody (1:200, Fine Test, FNab00491), GLP1R Rabbit pAb (1:200, Bioss, bs-1559R). Followed by mixture with secondary antibody (Proteintech) at home temperature for 1 h. The staining results were analyzed using ImageJ software. The expression levels of ANPEP, APOB, and GLP1R were assessed via semiquantitative analysis by calculating the average optical density (AOD) of positive staining. The AOD was determined by dividing the integral optical density (IOD) by the stained area.

### Statistical analysis

All analyses were conducted using R programming language (version 3.6.0), with the Wilcoxon test applied to compare data between different groups. A *p*-value of less than 0.05 was considered statistically significant, unless otherwise specified.

## Results

### Results of sequencing data

In this study, lncRNA sequencing was performed on 15 human samples using the Illumina Novaseq high-throughput sequencing platform. A total of 1,280,983,532 raw reads were obtained, with a data volume of 192.08 Gb. After rigorous quality control procedures, including the removal of splice junctions and low-quality reads, 1,202,416,476 clean reads were retained, corresponding to a total of 180.02 Gbps of data. The Q20 values for these data were approximately 94.61%, and the Guanine-Cytosine (GC) content was approximately 56.64%, indicating high-quality sequencing. Furthermore, the alignment rate of all samples to the human genome exceeded 85%, further validating the reliability of the sequencing data (Table 1).

**Table 1:** mRNA and lncRNA sequencing data and alignment rate statistics

Sample	Raw reads	Raw bases	Clean reads	Clean bases	Error rate (%)	Q20 (%)	Q30 (%)	GC content (%)
LZZK0A1	84,786,384	12.71G	81,708,128	11.92G	0.03	97.79	96.15	58.13
LZZK0A2	80,761,460	12.11G	77,395,924	11.61G	0.03	95.15	91.67	53.25
LZZK0A3	79,236,926	11.88G	75,295,858	11.29G	0.03	96.19	93.60	53.17
LZZK0A4	86,028,170	12.9G	80,101,276	12.02G	0.03	95.68	92.69	58.18
LZZK0A5	81,162,956	12.17G	77,184,380	11.58G	0.03	96.10	93.54	54.68
LZZK0B1	81,181,236	12.17G	76,691,550	11.5G	0.03	94.81	90.95	56.81
LZZK0B2	91,073,598	13.66G	86,142,504	12.92G	0.04	94.26	89.87	56.76
LZZK0B3	87,793,658	13.16G	83,584,824	12.54G	0.04	93.31	88.25	57.06
LZZK0B4	82,276,192	12.34G	77,089,372	11.56G	0.04	94.05	89.70	54.19
LZZK0B5	105,513,888	15.82G	97,674,782	14.65G	0.04	93.84	89.78	59.04
LZZK0C1	95,071,942	14.26G	87,007,382	13.05G	0.05	92.66	87.21	58.88
LZZK0C2	84,472,168	12.67G	77,140,532	11.57G	0.03	94.77	91.42	60.59
LZZK0C3	89,635,550	13.44G	82,932,290	12.44G	0.03	94.76	91.37	54.27
LZZK0C4	75,753,848	11.36G	71,047,342	10.66G	0.04	93.07	88.08	57.63
LZZK0C5	76,235,556	11.43G	71,420,332	10.71G	0.04	92.79	87.58	57.00

### Biological pathways differed significantly among the three groups

**Fig. 1:**
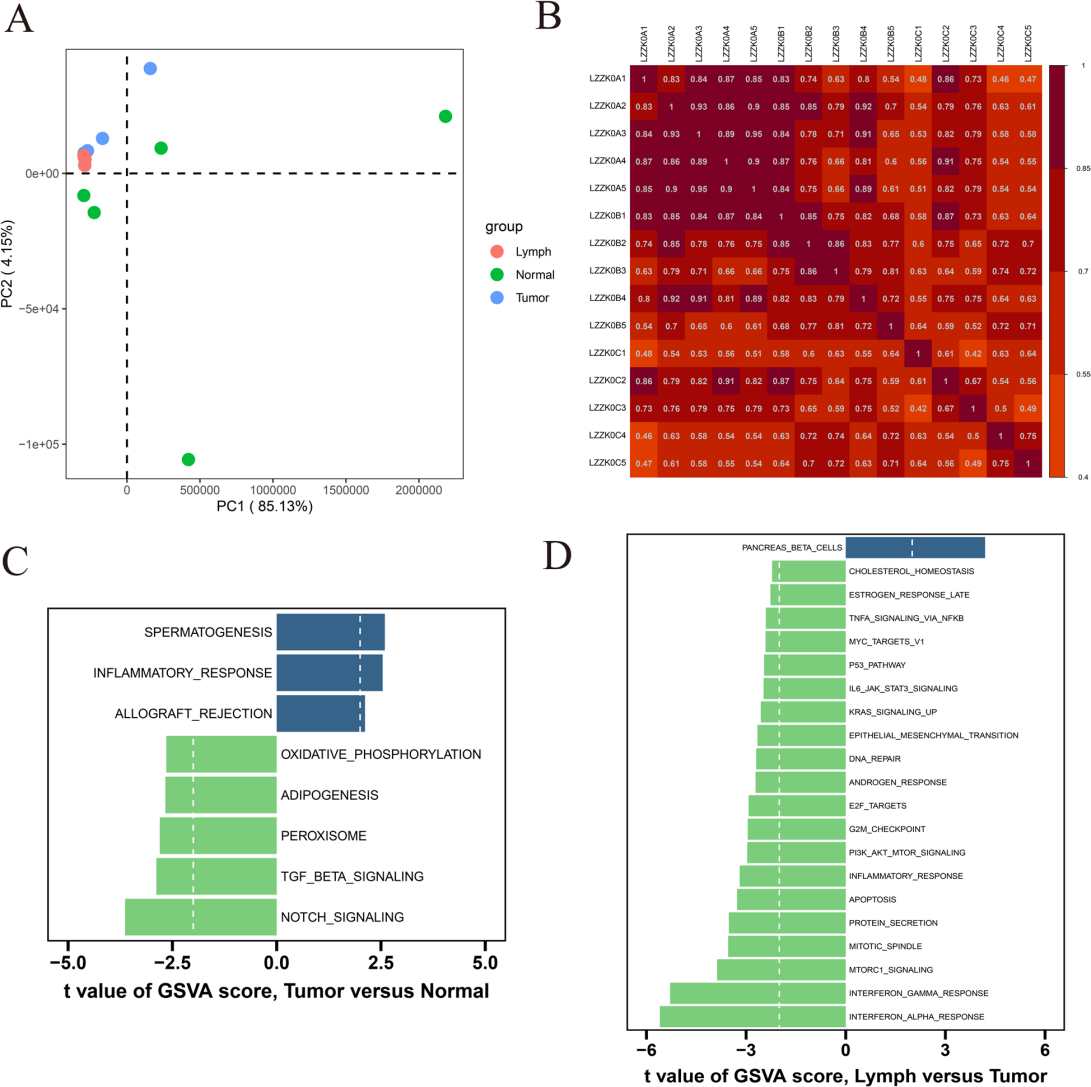
Biological pathways among control, primary, and metastatic groups. **A** PCA analysis. **B** A heatmap showing the correlation among the control, primary, and metastatic groups. **C** Differential biological pathways between the control and primary groups. **D** Differential biological pathways between the primary and metastatic groups

The assessment of the sequencing data quality through principal component analysis (PCA) of mRNA indicated good reproducibility within the control, primary, and metastatic groups. Notably, significant differences were observed among these groups (Fig. 1A, B). GSVA results revealed that pathways such as the ‘P53 pathway’ and ‘PI3K AKT mTOR signaling’ did not show significant differences between the primary and control groups; however, they were suppressed in the metastatic compared to the primary groups (Fig. 1C, D).

### Acquisition of DE-mRNAs, DE-miRNAs, and DE-lncRNAs between groups

**Fig. 2:**
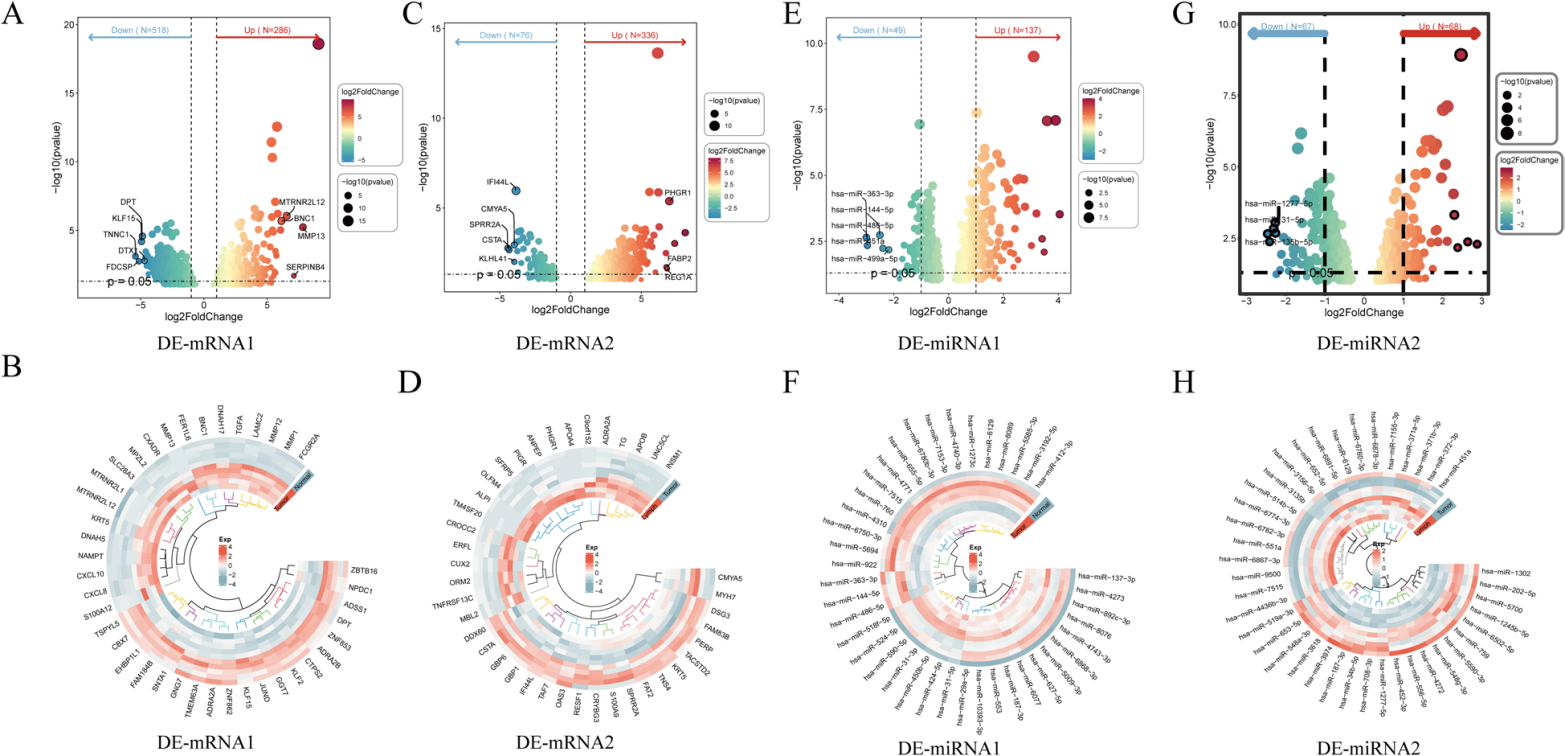
Identification of differentially expressed DE-mRNAs and DE-miRNAs. **A**, **E** Volcano plots showing DE mRNAs and DE miRNAs with up- and down-regulation between primary and control groups. **C**, **G** Volcano plots showing DE mRNAs and DE miRNAs with up- and down-regulation between the metastatic and primary groups. **B**, **F** Heatmaps of DE mRNAs and DE miRNAs between the primary and control groups. **D**, **H** Heatmaps of DE mRNAs and DE miRNAs between the metastatic and primary groups

**Fig. 3:**
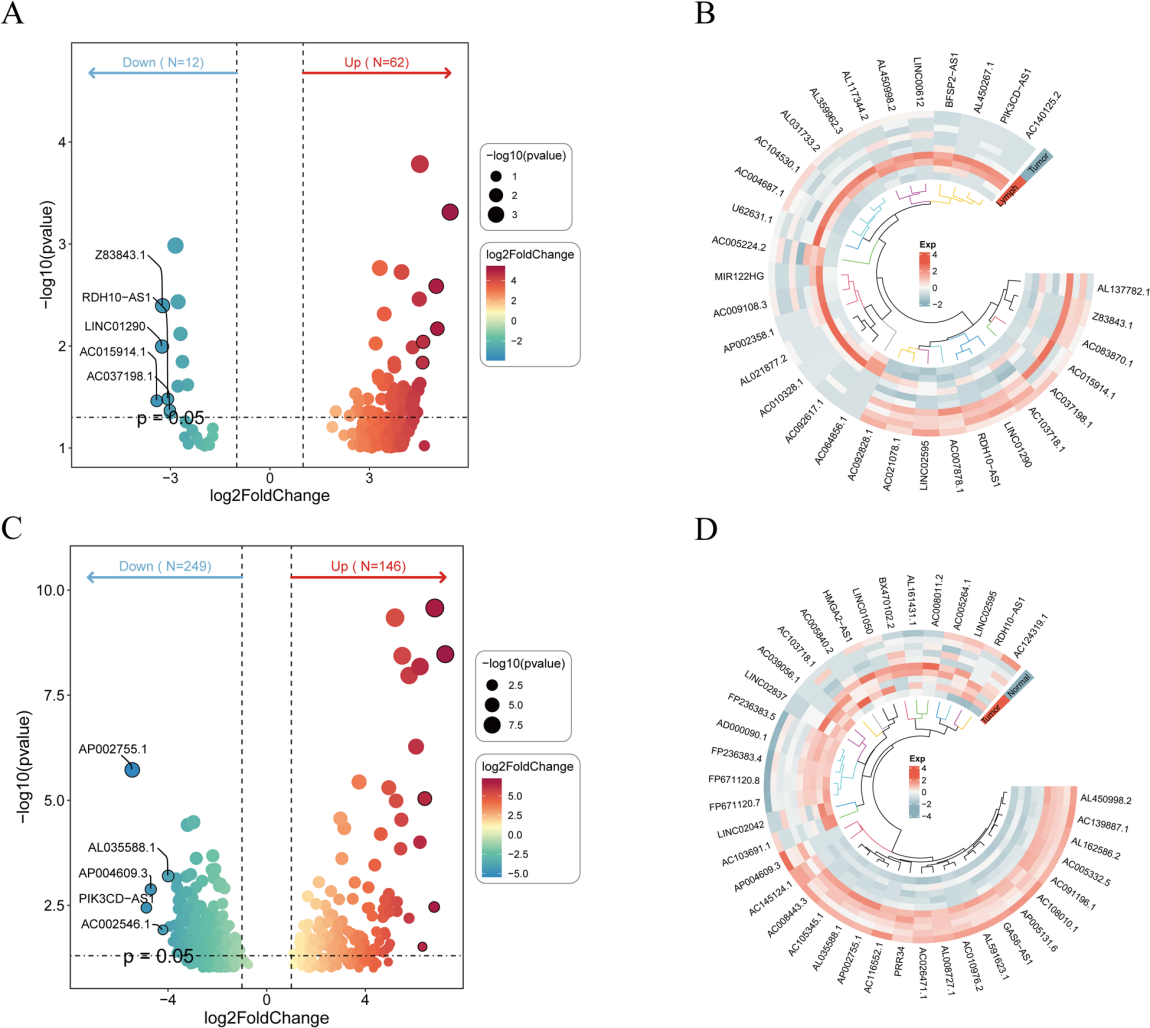
Identification of differentially expressed DE-lncRNAs. **A** Volcano plots showing DE-lncRNAs with up- and down-regulation between the primary and control groups. **B** Heatmaps of DE-lncRNAs between the primary and control groups. **C** Volcano plots showing DE-lncRNAs with up- and down-regulation between the metastatic and primary groups. **D** Heatmaps of DE-lncRNAs between the metastatic and primary groups

A total of 804 DE-mRNAs (DE-mRNA1) were identified between the primary and control groups, with 286 up-regulated and 518 down-regulated mRNAs (Fig. 2A, B). Between the metastatic and primary groups, 412 DE-mRNAs (DE-mRNA2) were detected, of which 336 were up-regulated and 76 down-regulated (Fig. 2C, D). Additionally, 186 DE-miRNA1 were found between the primary and control groups, comprising 137 up-regulated and 49 down-regulated miRNAs (Fig. 2E, F). Between the metastatic and primary groups, 135 DE-miRNA2 were identified, with 68 up-regulated and 67 down-regulated miRNAs (Fig. 2G, H). Furthermore, 395 DE-lncRNA1 were screened between the primary and control groups, including 146 up-regulated and 249 down-regulated lncRNAs (Fig. 3A, B). Between the metastatic and primary groups, 74 DE-lncRNA2 were identified, with 62 up-regulated and 12 down-regulated lncRNAs (Fig. 3C, D).

### Functional enrichment of a total of 446 candidate genes

**Fig. 4:**
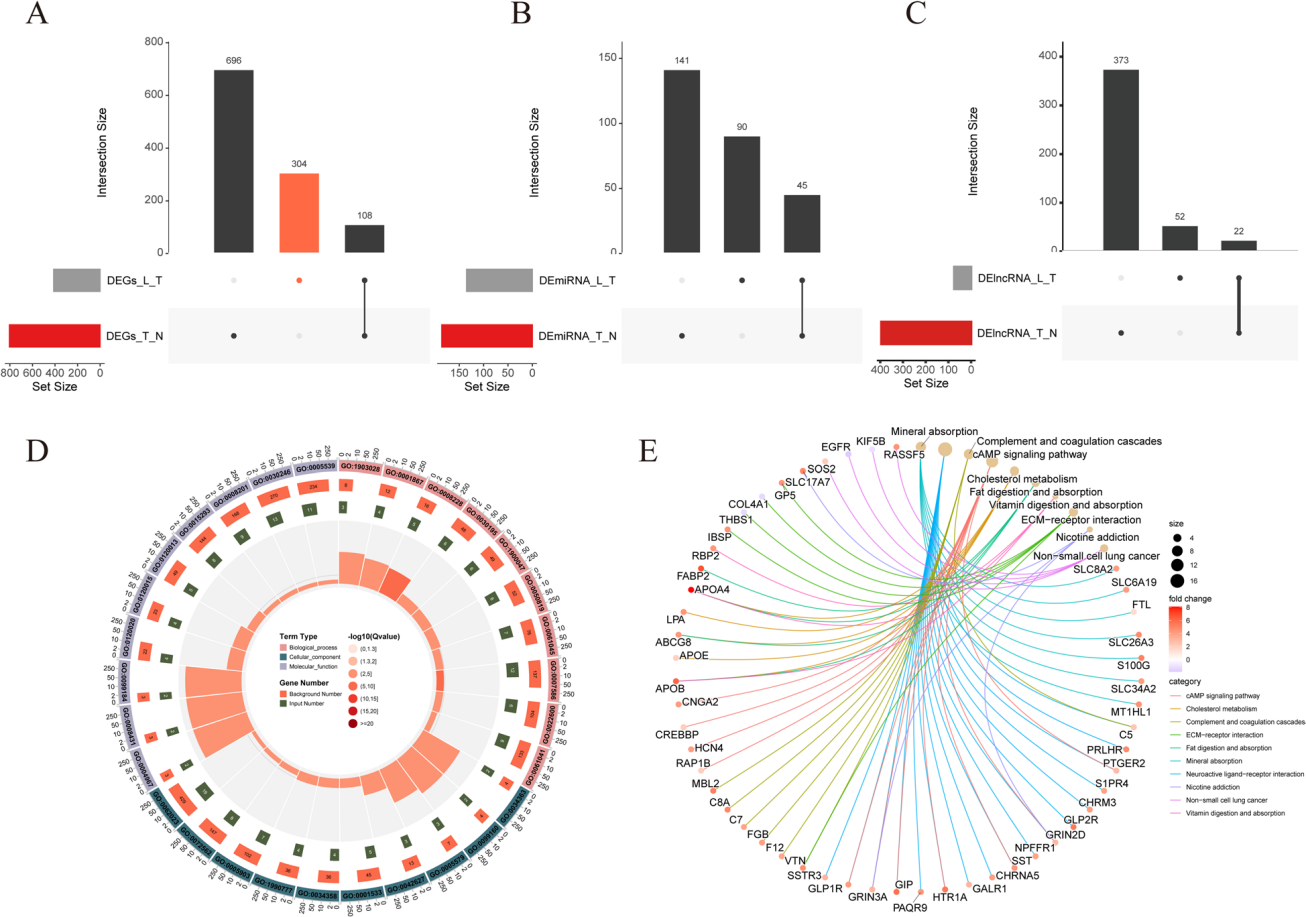
Differential gene distribution between groups and enrichment of candidate genes. **A**–**C** Candidate DE-mRNAs, DE-miRNAs, and DE-lncRNAs screened in DE-mRNA1 and DE-mRNA2. **D** GO enrichment of candidate genes. **E** KEGG pathways involved in the candidate genes

In order to identify differentially expressed mRNAs involved in the lymphatic metastasis process, differentially expressed genes specific to the metastasis group were defined as candidate genes for subsequent analysis. The analysis identified a total of 304 candidate genes from DE-mRNA1 and DE-mRNA2 (Fig. 4A), 90 candidate DE-miRNAs from DE-miRNA1 and DE-miRNA2 (Fig. 4B), and 52 candidate DE-lncRNAs from DE-lncRNA1 and DE-lncRNA2 (Fig. 4C). The Gene Ontology (GO) enrichment analysis revealed that these candidate genes were associated with biological functions such as ‘digestive system process’, ‘intestinal lipid absorption’, and ‘negative regulation of wound healing’ (Fig. 4D). The KEGG pathway analysis highlighted pathways like ‘mineral absorption’, ‘cholesterol metabolism’, and ‘cAMP signaling pathway’ as being significantly involved (Fig. 4E).

### Demonstration of lncRNA-miRNA-mRNA regulatory network

**Fig. 5:**
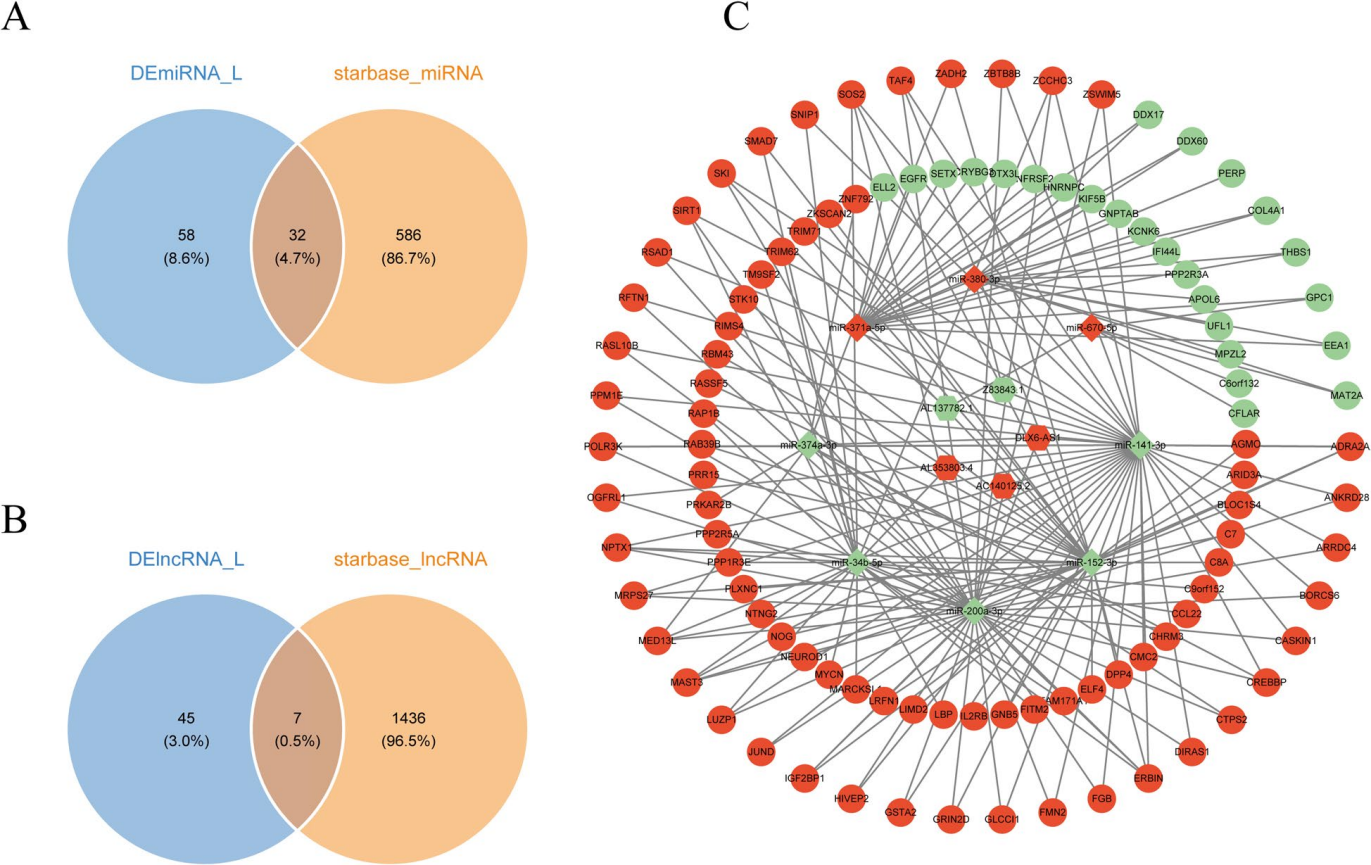
LncRNA-miRNA-mRNA co-regulatory network. **A** Venn diagram showing the overlap between candidate miRNAs and miRNAs predicted by the database. Target miRNAs. **B** Venn diagram showing the overlap between candidate lncRNAs and lncRNAs predicted by the database. **C** The molecular regulatory network of OSCC

A total of 32 target miRNAs were identified by intersecting the 90 candidate miRNAs with 618 predicted miRNAs from the database (Fig. 5A). Similarly, 7 target lncRNAs were identified from the overlap of 52 candidate lncRNAs and 1443 predicted lncRNAs (Fig. 5B). Based on this data, a molecular regulatory network for OSCC was constructed (Fig. 5C). The lncRNA-miRNA-mRNA regulatory network revealed 8 miRNAs (e.g., miR-371a-5p, miR-380-3p, and miR-141-3p) and 5 corresponding lncRNAs (e.g., AL137782.1, Z83843.1, AL353803.4, AC140125.2, and DLX6-AS1) that were predicted to be part of this network (Fig. 5C).

### ANPEP, APOB, GLP1R, and SI were identified as biomarkers of OSCC

**Fig. 6:**
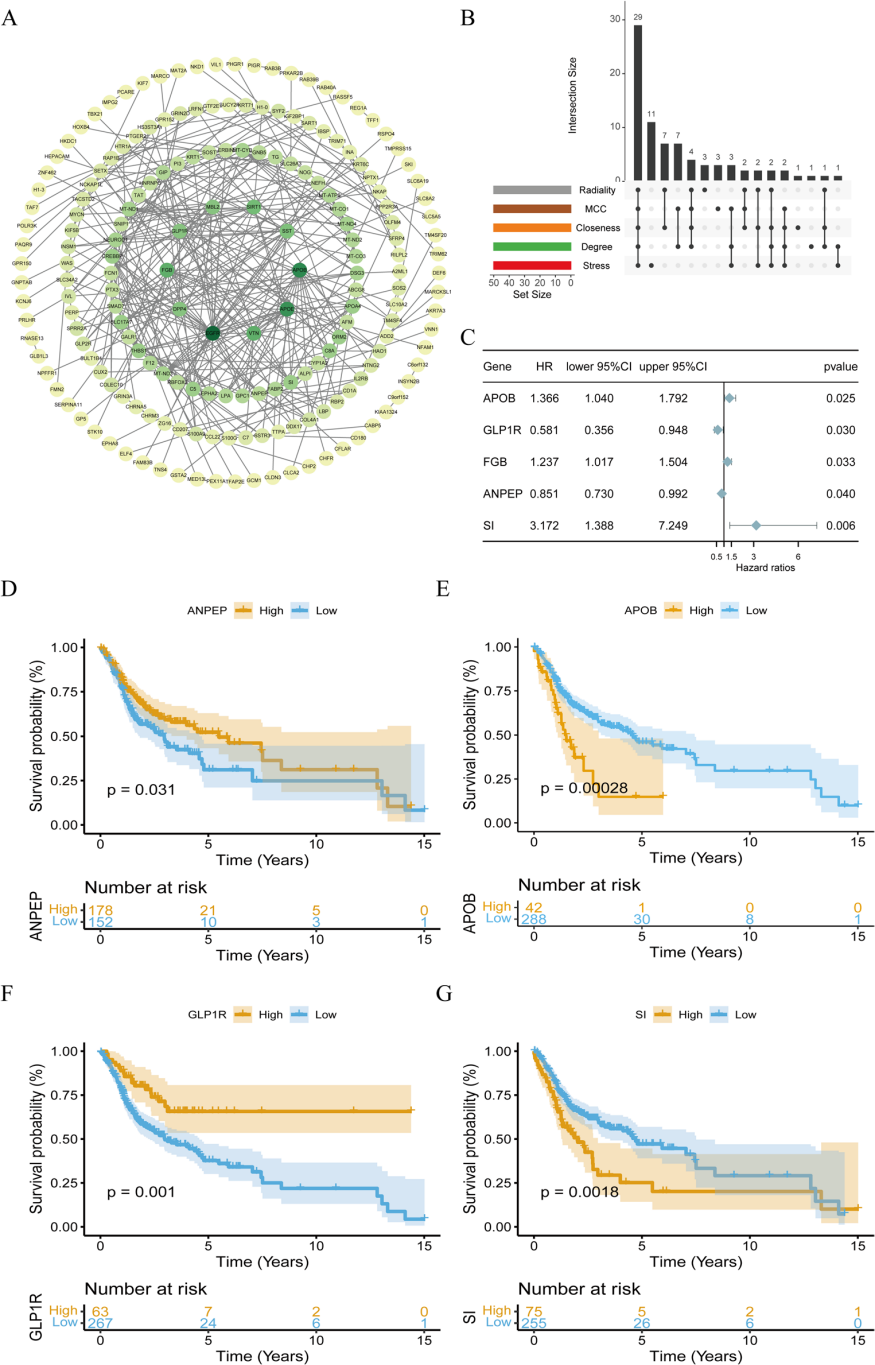
PPI network, potential biomarkers, and Kaplan–Meier analysis. **A** PPI network construction. The thickness of edges represents the combined score, while node color and size indicate the degree of interaction. **B** Potential biomarkers for OSCC. **C** The univariate Cox forest chart of prognosis-related genes. **D**–**G** Kaplan–Meier analysis

To explore the network interaction relationships of candidate genes at the protein level, the STRING (http://string-db.org) was utilized to construct a protein–protein interaction (PPI) network of the candidate genes. The PPI network showed strong interactions between EGFR, APOB, and APOE at the protein level (Fig. 6A). Based on the results of five algorithms (Degree, Maximal Clique Centrality (MCC), Closeness, Radality, Stress), 29 potential biomarkers for OSCC were screened (Fig. 6B). Among these, ANPEP, APOB, GLP1R, SI, and FGB were found to be prognosis-related genes for OSCC (*p* < 0.05) (Fig. 6C). In TCGA-OSCC, for OSCC patients with survival information, they were divided into high-expression and low-expression groups. K–M analysis revealed that biomarkers such as ANPEP, APOB, GLP1R, and SI showed significant differences in survival between the high and low expression groups (Fig. 6D–G). Specifically, ANPEP and GLP1R were found to be protective factors for OSCC, while APOB and SI were risk factors for OSCC.

### Differential expression of biomarkers in different clinical features

**Fig. 7:**
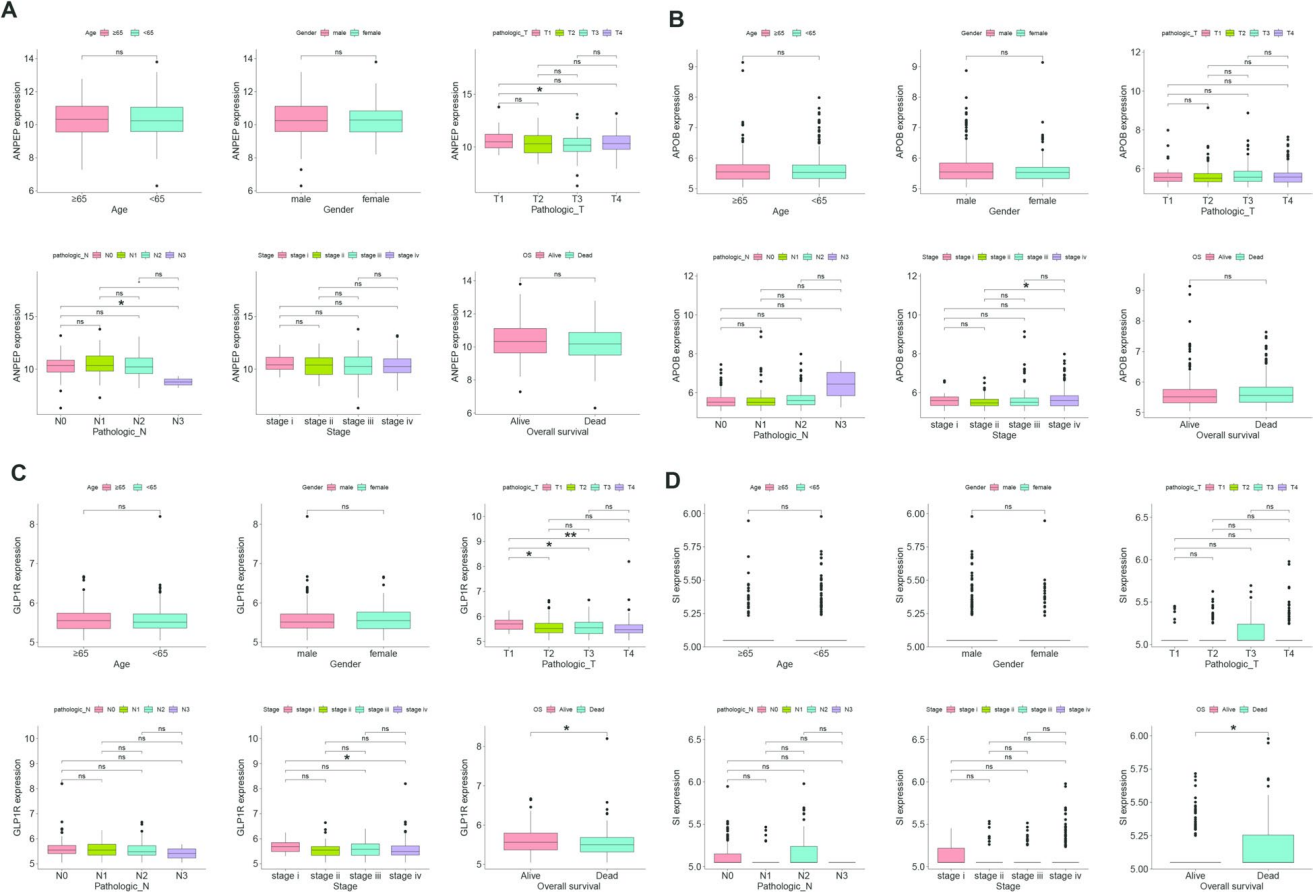
Differential expression of biomarkers across various clinical features. **A** ANPEP, **B** APOB, **C** GLP1R, **D** SI. ns: not significant, *p < 0.05, **p < 0.01

To analyze the relationship between biomarkers and clinical features, in TCGA-OSCC, OSCC patients were divided into different clinical subgroups according to different clinical features. The results showed that ANPEP expression varied significantly in pathological T1, T3, and pathological N0 and N3 (Fig. 7A). APOB exhibited significant expression differences only at a specific stage (Fig. 7B). GLP1R expression differed significantly between stages, overall survival (OS) and pathological T stage, with significantly higher expression in OSCC stage IV than OSCC stage I (Fig. 7C). SI expression also showed significant variation in overall survival (Fig. 7D).

### SI and APOB had the relatively highest mutation rates

**Fig. 8:**
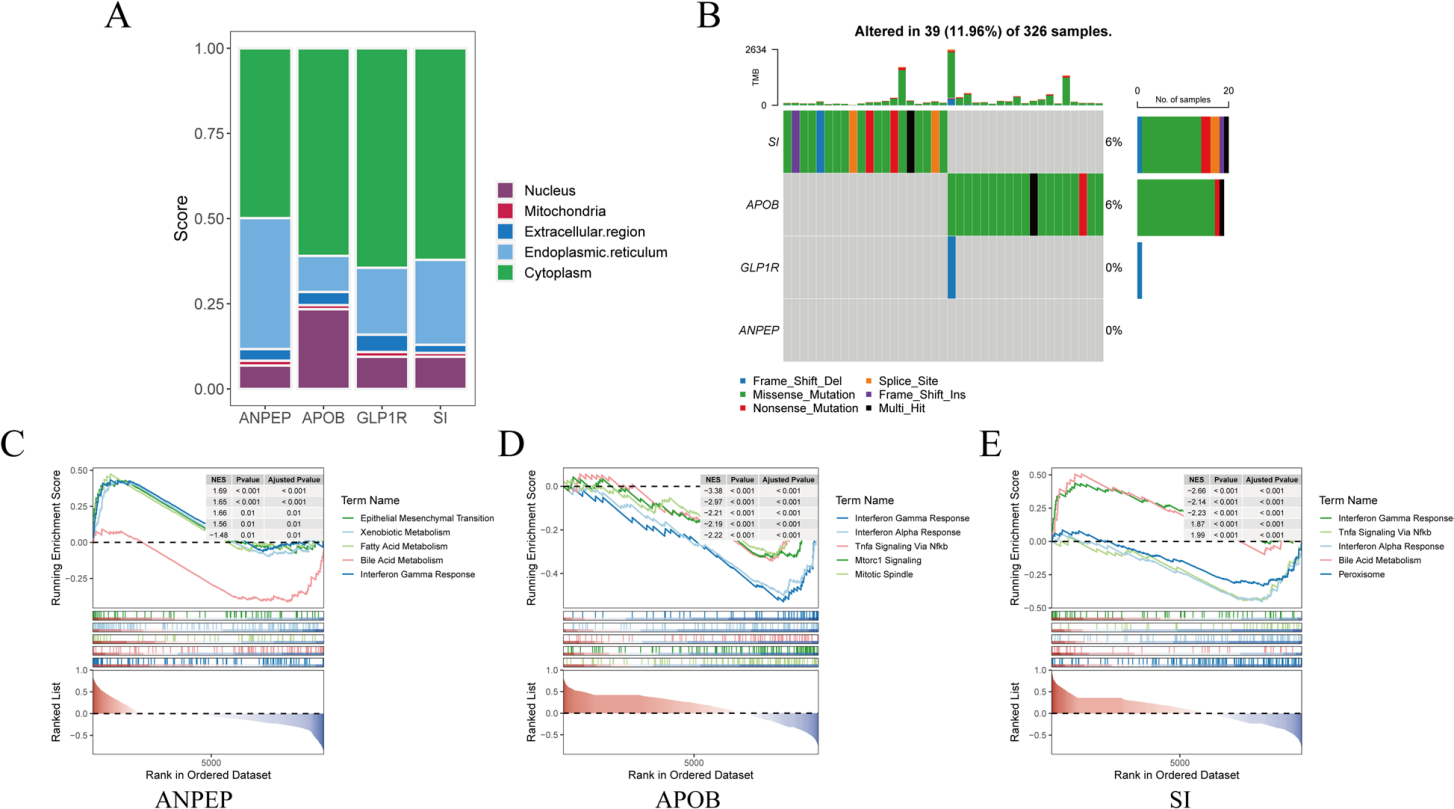
Subcellular localization, mutation profile, and biological function. **A** Subcellular localization analysis, **B** Biomarker mutation profile, **C**–**E** Biological functions co-enriched by ANPEP, APOB, and SI

The mRNALocater database was utilized to analyze the locations of the biomarkers within cells. Subcellular localization analysis revealed that the biomarkers were predominantly expressed in the cytoplasm, followed by notable expression in the nucleus and endoplasmic reticulum, with relatively low expression in the mitochondria (Fig. 8A). The "maftools" package was used to analyze the mutation status of biomarkers in TCGA-OSCC samples through the TCGA database. Mutation analysis identified SI and APOB as having the highest mutation rates in OSCC samples, with APOB primarily exhibiting missense mutations, while SI displayed multiple mutation types (Fig. 8B). Additionally, to elucidate the potential biological functions of biomarkers in cancer metastasis, a Gene Set Enrichment Analysis (GSEA) was performed on the sequencing data to explore the potential functions of the biomarkers. The results showed that the ‘interferon-gamma response’ biological function was co-enriched by ANPEP, APOB, and SI (Fig. 8C–E).

### BIBW2992 and PF.02341066 were key drugs for OSCC

**Fig. 9:**
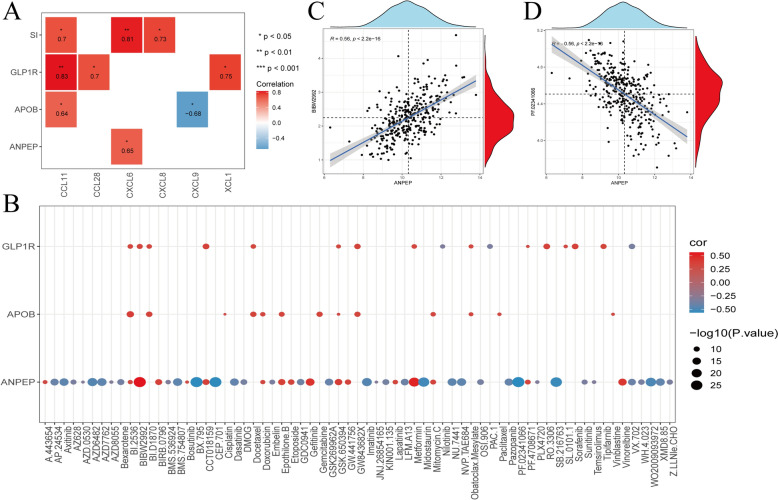
Immunological features and correlation analysis between biomarkers and drug sensitivity. **A** Correlation analysis between biomarkers, **B** Bubble plots of IC_50_ correlations of biomarkers with drugs, **C** Correlation between BIBW2992 (Afatinib) and ANPEP, **D** Correlation between PF.02341066 (Crizotinib) and ANPEP

In the sequencing training set (metastatic group and primary group), Spearman correlation was adopted to analyze the correlations between biomarkers and different immune features. Immunological analysis showed significant positive correlations between APOB (cor = 0.64), GLP1R (cor = 0.83), and SI (cor = 0.70) with CCL11. SI also showed significant positive correlations with CXCL6 (cor = 0.81) and CXCL8 (cor = 0.73) (*p* < 0.05). APOB was negatively correlated with CXCL9 (cor = -0.68), while GLP1R was positively correlated with CCL11 (cor = 0.83) and XCL1 (cor = 0.75) (*p* < 0.05) (Fig. 9A). Furthermore, in TCGA-OSCC, the pRRophetic R package was used to calculate the IC50 values of common chemotherapy and molecular targeted drugs for OSCC patients. Spearman correlation was employed to analyze the correlation between biomarkers and drug sensitivity. Bubble plots demonstrated the IC_50_ correlation of biomarkers with drugs (Fig. 9B). Among these, BIBW2992 (afatinib) and PF.02341066 (crizotinib) exhibited the highest correlation coefficients between IC_50_ and ANPEP, and were identified as key drugs for OSCC (Fig. 9C, D).

### Expression in the biomarker transfer group was significantly upregulated

**Fig. 10:**
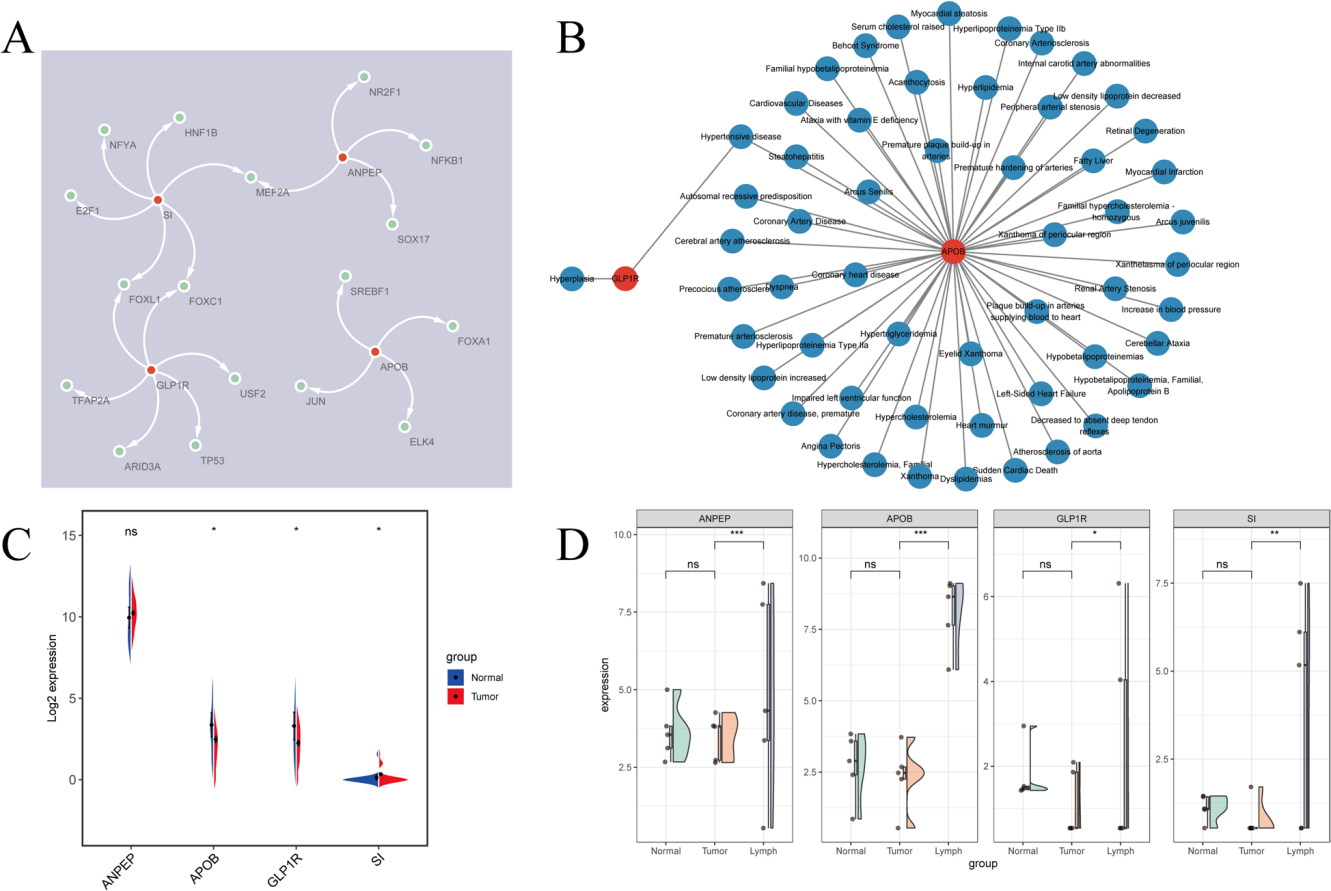
Regulatory network and biomarker expression across three groups. **A** Analysis of the transcription factor regulatory network, **B** Diseases associated with biomarkers, **C** Expression of biomarkers between primary and control groups, **D** Expression of biomarkers across primary, control, and metastatic groups. ns: not significant, *p < 0.05, **p < 0.01, ***p < 0.001

A total of 17 TFs were predicted based on biomarkers, with FOXL1 and FOXC1 identified as TFs for GLP1R and SI. MEF2A was the sole TF shared between SI and ANPEP (Fig. 10A). In the biomarker-disease co-expression network, APOB was associated with the highest number of diseases significantly linked to OSCC, while GLP1R predicted only two diseases, including hypertensive disease, which was co-predicted by both APOB and GLP1R (Fig. 10B). Additionally, expression trends of biomarkers in the TCGA-OSCC dataset for the primary and control groups differed from those observed in the sequencing data, as the latter focused primarily on the metastatic and primary groups (Fig. 10C). Finally, in the sequencing data, the Wilcoxon rank-sum test was used to compare the expression levels of biomarkers among different groups. The results showed that the expression of the biomarker was relatively higher in the metastatic group (Fig. 10D).

### Verification of the novel biomarkers by qRT-PCR and immunohistochemistry

**Fig.11:**
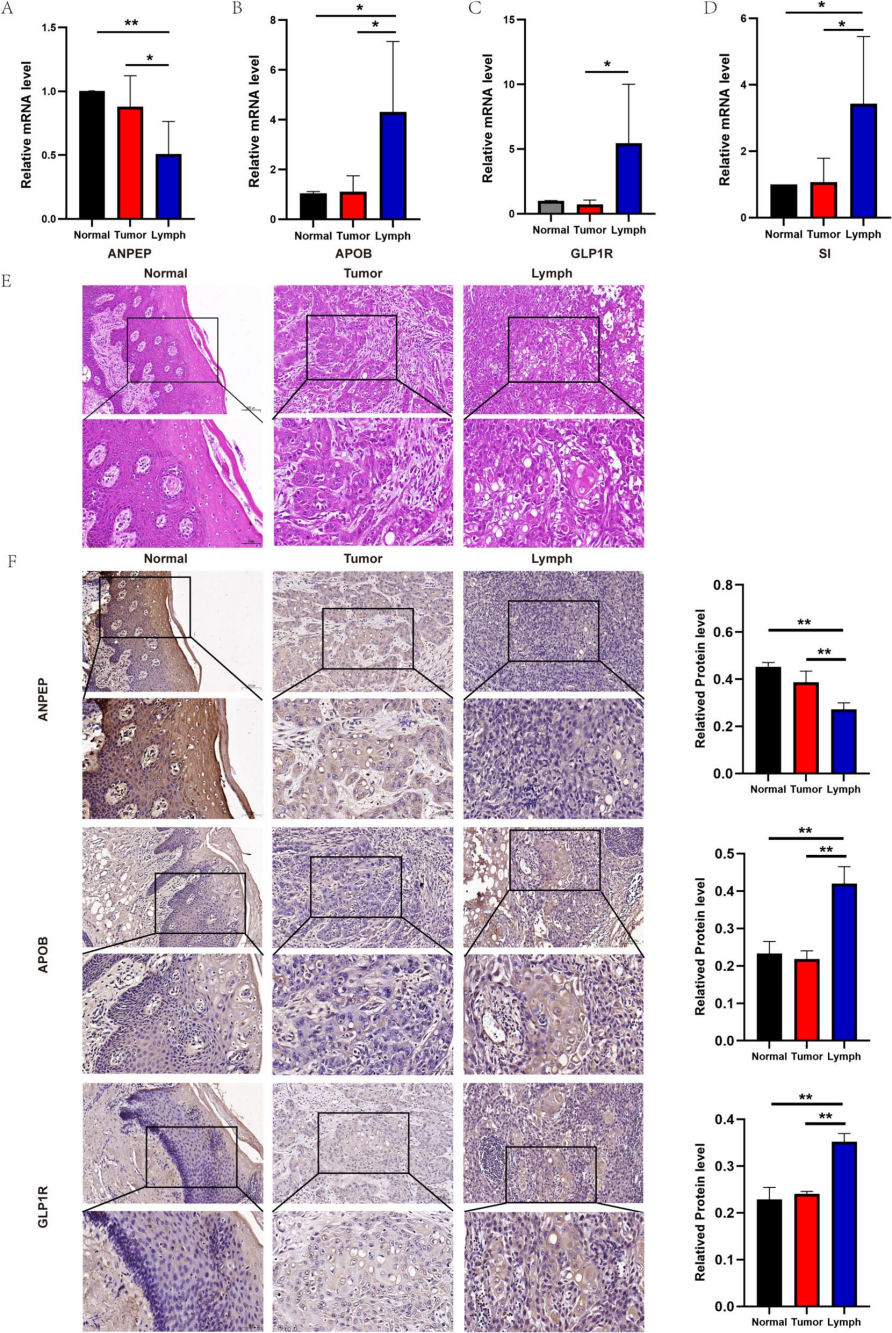
qRT-PCR and immunohistochemistry validation of the expression levels of key biomarkers. **A**–**D** Relative mRNA expression of ANPEP, APOB, GLP1R and SI. **E** Hematoxylin and eosin (H&E) staining of three groups. **F** Immunohistochemical staining for ANPEP, APOB, and GLP1R in three groups and the relevant statistical diagram of immunohistochemical results. Images were taken under × 200 and × 400 magnifications for each field. *p < 0.05, **p < 0.01

To validate the results of RNA-seq and bioinformatics analysis, we quantified the expression levels of ANPEP, APOB, GLP1R, and SI using qRT-PCR. The validation assay results indicated that the changes in the expression of ANPEP, APOB, GLP1R, and SI were consistent with the RNA-seq data (*p* < 0.05) (Fig. 11A–D). H&E staining confirmed the samples as squamous cell carcinoma, with metastasis present in the lymph node tissues (Fig. 11E). We also assessed the protein expression of ANPEP, APOB, and GLP1R in tissues from the primary, tumor, and metastatic groups using IHC staining. The IHC results showed that ANPEP staining intensity was strong in the primary group and mild in the metastatic group. APOB and GLP1R exhibited the highest expression levels in the metastatic group (Fig. 11F). The IHC staining results confirmed that the expression patterns of ANPEP, APOB, and GLP1R were consistent with the RNA-seq data.

## Discussion

Oral Squamous Cell Carcinoma (OSCC), characterized by high morbidity and mortality, constitutes over 90% of oral cancers [1, 18]. Genetic events and molecular mechanisms have been widely recognized as having certain effects on the initiation, progression, and metastasis of tumors [19–21]. Previous studies have identified HSPA5, HYOU1, and PDIA4 as participants in the integrin-related protein network of OSCC through Next-Generation Sequencing and bioinformatics methods [22]. This study conducted a comprehensive transcriptome analysis of adjacent cancer tissues and primary and metastatic OSCC samples, identifying differentially expressed messenger RNAs (mRNAs), microRNAs (miRNAs), and long non-coding RNAs (lncRNAs). Additionally, the mutations, functions, and potential drug associations of these molecules were explored. The findings revealed that ANPEP, APOB, GLP1R, and SI exhibited significant survival differences in Kaplan–Meier (KM) curves, with APOB and SI showing the highest mutation rates. Immunological analysis indicated that there is a significant positive correlation between APOB, GLP1R, and CCL11. SI was not only significantly positively correlated with CCL11 but also showed significant positive correlations with CXCL6 and CXCL8. APOB exhibited a negative correlation with CXCL9, while GLP1R demonstrated positive correlations with CCL11 and XCL1. Particularly noteworthy was the strongest correlation observed between BIBW2992 (afatinib) and PF.02341066 (crizotinib) and these biomarkers.

Aminopeptidase N (ANPEP), also known as APN/CD13, has been implicated in various tumors [23–25]. It is involved in tumor cell proliferation, differentiation, migration, angiogenesis, invasion, and metastasis [26, 27]. Pharmacological inhibitors targeting APN/CD13 have demonstrated efficacy in reducing tumor growth and progression in APN/CD13-positive tumors [28, 29]. Additionally, these APN/CD13 inhibitors are relatively effective in the early stages of tumorigenesis and can inhibit tumor growth [30]. Apolipoprotein B (APOB), a member of the apolipoprotein family, is linked to several cancers, including non-small cell lung cancer [31], hepatocellular carcinoma [32, 33], breast cancer [34], colorectal carcinoma [35], and gallbladder cancer [36]. Despite these associations, the underlying mechanisms remain unclear. Genetic variants of APOB and CDH13 in the cholesterol pathway have been shown to affect survival in non-small cell lung cancer by modulating gene expression [31]. Consistent with these studies, APOB was found to be upregulated in OSCC [37], aligning with our findings. Glucagon-like peptide-1 receptor (GLP1R), a seven-transmembrane protein encoded by the GLP1R gene, is upregulated in endometrial cancer (EC) and promotes proliferation and metastasis, thereby accelerating EC progression [38]. Additionally, GLP1R agonist Liraglutide has been shown to inhibit proliferation and migration in thyroid cancer cells via the PI3K/Akt/mTOR pathway [39]. Sulfite oxidase (SI) encodes a sucrase-isomaltase enzyme expressed in the intestinal brush border. Previous studies have linked SI to carcinogenesis in human lung adenocarcinoma cells [40].

However, the roles of ANPEP, APOB, GLP1R, and SI in OSCC have not been deeply explored. The results of this study suggested that ANPEP, APOB, GLP1R, and SI could potentially serve as biomarkers for OSCC, expanding their application scope in the field of oncology to some extent, and also providing new ideas for the diagnosis and treatment of OSCC. They are expected to become novel biomarkers for early detection and prognosis of OSCC. They have potential in early detection and may serve as serum or tissue markers. When combined with existing detection technologies, they can help improve the sensitivity and accuracy of early diagnosis of OSCC to a certain extent. For example, in high-risk populations such as long-term smokers and drinkers, HPV-infected individuals, screening for their expression levels can help detect potential tumor changes at an early stage. In terms of prognostic evaluation, it may be possible to develop a more accurate prognostic prediction model based on the expression status of these biomarkers. Physicians can then more precisely assess the risk of recurrence and survival probabilities for patients, leading to the formulation of personalized treatment plans.

Numerous studies have reported the involvement of lncRNA-miRNA-mRNA regulatory networks in tumor metastasis. For instance, lncRNA LIFR-AS1 sponges miR-29a to upregulate NFIA, thereby promoting osteosarcoma cell proliferation [41]. A previous study identified the lncRNA-miRNA-mRNA axis involving LINC01140, miR-140-5p, and FGF9, which regulates gastric cancer phenotypes and impacts cell aggressiveness [42]. Additionally, the DE-lncRNAs AP002498.1 and the LINC01871/miR-4644 and miR-185-5p/GNLY axes were shown to be significantly linked to distant metastasis in colorectal adenocarcinoma [43]. Another study demonstrated that circ-LRP6 regulates the miR-141-3p/HDAC4/HMGB1 axis to contribute to osteosarcoma progression [44]. In this study, a similar lncRNA-miRNA-mRNA regulatory network was identified in OSCC. Five lncRNAs (AL137782.1, Z83843.1, AL353803.4, AC140125.2, and DLX6-AS1) were associated with eight miRNAs (miR-371a-5p, miR-380-3p, miR-670-5p, miR-141-3p, miR-152-3p, miR-200a-3p, miR-34b-5p, and miR-374a-3p) in OSCC. The lncRNA distal-less homeobox 6 antisense 1 (DLX6-AS1) has been implicated in the pathogenesis of multiple cancers [45, 46]. For instance, the DLX6-AS1/miR-16-5p axis has been shown to regulate autophagy and apoptosis in non-small cell lung cancer [47]. These lncRNAs may contribute to OSCC progression via the lncRNA-miRNA-mRNA axis. miR-141-3p and miR-200a-3p, members of the miR-200 family, modulate EMT by regulating epithelial (E-)cadherin expression [48]. The downregulation of the miR-200 family, including miR-141-3p, has been observed in OSCC [49]. Supporting these findings, Mingguo Cao et al. [50] demonstrated that upregulation of miR-141-3p inhibits OSCC cell invasion, proliferation, and migration by targeting PBX1 through the JAK2/STAT3 pathway. These results highlight the significant role of the lncRNA-miRNA-mRNA axis in the progression and metastasis of OSCC. However, current findings indicate that lncRNAs and miRNAs had been more focused on mesenchymal tumor types such as osteosarcoma in previous studies, but OSCC, as an epithelial tumor, still exhibits biological differences from mesenchymal tumors [51]. Therefore, in the future, we will delve deeper into the role of the lncRNA-miRNA-mRNA axis in the progression and metastasis of OSCC, revealing its molecular mechanisms to provide a more solid theoretical basis for therapeutic strategies.

Additionally, a total of 17 Transcription Factors (TFs) were predicted based on the biomarkers, among which FOXL1 and FOXC1 were identified as TFs predicted by GLP1R and SI. The Fork head box C1 (FOXC1), a Forkhead box protein overexpressed in various malignancies, binds to DNA to regulate gene expression [52]. It has been shown to be upregulated in OSCC, and its knockdown inhibits cell proliferation and migration [53]. Furthermore, lncRNA LINC01929 has been reported to accelerate OSCC progression by targeting the miR-137-3p/FOXC1 axis [54]. Other TFs, such as Forkhead Box L1 (FOXL1), may also serve as important targets and predictive biomarkers for OSC.

Previous studies have shown that afatinib can target the EGFR/mTOR/Mcl-1 axis, inducing tumor cell apoptosis, and has the potential to become a treatment strategy for oral cancer [55]. Additionally, it is an effective first-line drug for the treatment of locally advanced or metastatic non-small cell lung cancer (NSCLC) [56]. It also demonstrates a better response rate in patients with local or metastatic recurrence after cisplatin treatment [57]. Crizotinib, on the other hand, inhibits tumor progression by inhibiting ROS1 or ALK and has been approved for the treatment of advanced lung adenocarcinoma patients with c-ROS1 (ROS1) and anaplastic lymphoma kinase (ALK) gene rearrangements [58]. Moreover, crizotinib has also been found to target head and neck squamous cell carcinoma carrying MET point mutations (R1004) [59]. These studies indicate that afatinib and crizotinib can affect the occurrence and development of tumors through different mechanisms. Our research results suggest that afatinib and crizotinib are potential key drugs for the treatment of OSCC. This finding supports the application potential of these two drugs in OSCC, providing new targeted treatment strategies for OSCC patients.

Immunological studies have shown a significant positive correlation between APOB, GLP1R, and CCL11, revealing a potential biological pathway. CCL11, as a chemokine, plays a role in tumor cell migration, angiogenesis, and the regulation of immune cells [60]. This suggests that APOB and GLP1R may indirectly affect cell migration and immune cell infiltration in the tumor microenvironment by regulating CCL11, but further research is needed to confirm this.This suggests that APOB and GLP1R may indirectly affect cell migration and immune cell infiltration in the tumor microenvironment by regulating CCL11, but further research is needed to confirm this.APOB and GLP1R may indirectly affect cell migration and immune cell infiltration in the tumor microenvironment by regulating CCL11. CXCL6 and CXCL8 play significant roles in tumor progression and inflammatory responses [61]. The positive correlation between SI and these factors suggests that SI may be involved in the regulation of tumor-associated inflammation. CXCL9 enhances immune surveillance and promotes the killing of tumor cells [62], while the negative correlation between APOB and CXCL9 may imply a potential role of APOB in tumor immune evasion. Although these hypotheses require further validation, they provide important directions for future research and may offer new insights for the optimization of immunotherapy.

In conclusion, this study identified differentially expressed mRNAs, miRNAs, and lncRNAs between primary and control groups, as well as metastatic and primary groups, based on whole transcriptome sequencing data and bioinformatics analysis. ANPEP, APOB, GLP1R, and SI were recognized as potential biomarkers for OSCC, providing a valuable reference for further investigation. However, despite providing preliminary insights, there are some limitations that need to be addressed in future studies. First, the sample size of the study is relatively small, which may affect the statistical significance and general applicability of the results. Although the current data can reveal some meaningful patterns of differential expression, the limited sample size may lead to the neglect of certain key biomarkers and may not fully represent the heterogeneity of the OSCC patient population. Therefore, future studies should increase the sample size, including patients with OSCC from more regions, different clinical stages, and various pathological subtypes, to enhance the broad applicability and clinical reference value of the results. Additionally, considering the molecular heterogeneity of OSCC, future studies should also focus on individual differences and explore how to combine different biomarkers with patients' clinical characteristics. Second, while this study used bioinformatics analysis combined with qRT-PCR and immunohistochemistry experiments to validate potential biomarkers, some of the validation experiments still have limitations. For example, the immunohistochemistry experiment for SI was not completed, mainly due to the lack of SI antibodies, which affected the study of the specific expression patterns and functions of this molecule in OSCC. To further validate the role of SI and other candidate biomarkers in OSCC, future studies will employ experimental techniques such as Western Blot and immunohistochemistry to explore their protein expression. In addition, we plan to construct more precise OSCC cell lines and animal models to more comprehensively analyze the role of these molecules in tumor progression at the cellular and in vivo levels, further verifying their reliability as biomarkers. Furthermore, this study found a significant correlation between ANPEP and the IC50 values of drugs BIBW2992 (Afatinib) and PF.02341066 (Crizotinib), but the specific mechanisms of action between ANPEP and these drugs are not yet clear. Future research will need to systematically evaluate the regulatory effects of these two drugs on ANPEP and explore their potential mechanisms of influencing OSCC cell proliferation, migration, or drug resistance through ANPEP. Additionally, we need to consider the clinical translation of these drugs, design experimental models suitable for clinical use, and further verify the relationship between ANPEP and these drugs, in hopes of providing more practical molecular targets for clinical treatment. Despite these limitations, the identification of biomarkers in OSCC offers valuable insights for future research.

## Supplementary Information

Additional file 1.

## Data Availability

No datasets were generated or analysed during the current study.

## References

[1] Sung H, Ferlay J, Siegel RL, Laversanne M, Soerjomataram I, Jemal A, et al. Global cancer statistics 2020: GLOBOCAN estimates of incidence and mortality worldwide for 36 cancers in 185 countries. CA Cancer J Clin. 2021;71(3):209–49. 33538338 10.3322/caac.21660

[2] Omar E. Current concepts and future of noninvasive procedures for diagnosing oral squamous cell carcinoma—a systematic review. Head Face Med. 2015;11:6. 25889859 10.1186/s13005-015-0063-z PMC4396078

[3] Hoene G, Gruber RM, Leonhard JJ, Wiechens B, Schminke B, Kauffmann P, et al. Combined quality of life and posttraumatic growth evaluation during follow-up care of patients suffering from oral squamous cell carcinoma. Mol Clin Oncol. 2021;15(3):189. 34349989 10.3892/mco.2021.2351 PMC8327079

[4] Suresh GM, Koppad R, Prakash BV, Sabitha KS, Dhara PS. Prognostic indicators of oral squamous cell carcinoma. Ann Maxillofac Surg. 2019;9(2):364–70. 31909017 10.4103/ams.ams_253_18 PMC6933976

[5] Johnson DE, Burtness B, Leemans CR, Lui VWY, Bauman JE, Grandis JR. Head and neck squamous cell carcinoma. Nat Rev Dis Primers. 2020;6(1):92. 33243986 10.1038/s41572-020-00224-3 PMC7944998

[6] Tojo S, Nakashiro KI, Kuribayashi N, Uchida D. Serum CXCL13 as a novel biomarker in oral squamous cell carcinoma. Cancer Med. 2024;13(18): e70263. 39344390 10.1002/cam4.70263 PMC11440027

[7] Shaikh S, Basu S, Bag S, Chatterjee A, Datta S, Banerjee D, et al. Uracil as a biomarker for spatial pyrimidine metabolism in the development of gingivobuccal oral squamous cell carcinoma. Sci Rep. 2024;14(1):11609. 38773214 10.1038/s41598-024-62434-z PMC11109148

[8] Hashimoto T, Nakamura Y, Mishima S, Nakayama I, Kotani D, Kawazoe A, et al. Whole-transcriptome sequencing in advanced gastric or gastroesophageal cancer: a deep dive into its clinical potential. Cancer Sci. 2024;115(5):1622–33. 38429886 10.1111/cas.16109 PMC11093190

[9] Nagasaka M, Zhang SS, Baca Y, Xiu J, Nieva J, Vanderwalde A, et al. Pan-tumor survey of ROS1 fusions detected by next-generation RNA and whole transcriptome sequencing. BMC Cancer. 2023;23(1):1000. 37853341 10.1186/s12885-023-11457-2 PMC10585918

[10] Zhang Q, Zhang J, Jin H, Sheng S. Whole transcriptome sequencing identifies tumor-specific mutations in human oral squamous cell carcinoma. BMC Med Genom. 2013;6:28. 10.1186/1755-8794-6-28 PMC3844419 24007313

[11] Dharavath B, Butle A, Chaudhary A, Pal A, Desai S, Chowdhury A, et al. Recurrent UBE3C-LRP5 translocations in head and neck cancer with therapeutic implications. NPJ Precis Oncol. 2024;8(1):63. 38438481 10.1038/s41698-024-00555-4 PMC10912599

[12] Meng X, Lou QY, Yang WY, Wang YR, Chen R, Wang L, et al. The role of non-coding RNAs in drug resistance of oral squamous cell carcinoma and therapeutic potential. Cancer Commun (Lond). 2021;41(10):981–1006. 34289530 10.1002/cac2.12194 PMC8504146

[13] Wang WT, Han C, Sun YM, Chen TQ, Chen YQ. Noncoding RNAs in cancer therapy resistance and targeted drug development. J Hematol Oncol. 2019;12(1):55. 31174564 10.1186/s13045-019-0748-z PMC6556047

[14] Huang F, Xin C, Lei K, Bai H, Li J, Chen Q. Noncoding RNAs in oral premalignant disorders and oral squamous cell carcinoma. Cell Oncol (Dordr). 2020;43(5):763–77. 32495292 10.1007/s13402-020-00521-9 PMC12990699

[15] Monden N, Asakage T, Kiyota N, Homma A, Matsuura K, Hanai N, et al. A review of head and neck cancer staging system in the TNM classification of malignant tumors (eighth edition). Jpn J Clin Oncol. 2019;49(7):589–95. 31194232 10.1093/jjco/hyz052

[16] Bolger AM, Lohse M, Usadel B. Trimmomatic: a flexible trimmer for Illumina sequence data. Bioinformatics. 2014;30(15):2114–20. 24695404 10.1093/bioinformatics/btu170 PMC4103590

[17] Love MI, Huber W, Anders S. Moderated estimation of fold change and dispersion for RNA-seq data with DESeq2. Genome Biol. 2014;15(12):550. 25516281 10.1186/s13059-014-0550-8 PMC4302049

[18] Siegel RL, Giaquinto AN, Jemal A. Cancer statistics, 2024. CA Cancer J Clin. 2024;74(1):12–49. 38230766 10.3322/caac.21820

[19] Zhang C, Cai Q, Ke J. Poor prognosis of oral squamous cell carcinoma correlates with ITGA6. Int Dent J. 2023;73(2):178–85. 35820930 10.1016/j.identj.2022.05.010 PMC10023534

[20] Khromov T, Fischer L, Leha A, Bremmer F, Fischer A, Schliephake H, et al. Combined biomarker system predicts prognosis in patients with metastatic oral squamous cell carcinoma. Cancers (Basel). 2023;15(20):4924. 37894290 10.3390/cancers15204924 PMC10605069

[21] Chen Y, Gao Z, Mohd-Ibrahim I, Yang H, Wu L, Fu Y, et al. Pan-cancer analyses of bromodomain containing 9 as a novel therapeutic target reveals its diagnostic, prognostic potential and biological mechanism in human tumours. Clin Transl Med. 2024;14(2): e1543. 38303608 10.1002/ctm2.1543 PMC10835192

[22] Tseng CC, Tsou CH, Huang SY, Wu CW, Hsieh TH. Using next-generation sequencing and bioinformatic methods to predict new genes that may be regulated by CD47 in oral squamous cell carcinoma. Curr Issues Mol Biol. 2022;44(5):2243–56. 35678681 10.3390/cimb44050152 PMC9164064

[23] Liu Z, Yang Z, Xiong L, Li D, Zou Q, Yuan Y. ACO2 and ANPEP as novel prognostic markers for gallbladder squamous cell/adenosquamous carcinomas and adenocarcinomas. Int J Clin Oncol. 2020;25(7):1346–55. 32249333 10.1007/s10147-020-01651-8

[24] Goltsov AA, Maru DM, Katkhuda R, Duose DY, Luthra R, Correa AM, et al. ANPEP/CD13 expression as a marker of lymphovascular invasion and survival in esophageal adenocarcinoma. Ann Thorac Surg. 2024;118(1):241–51. 37806335 10.1016/j.athoracsur.2023.09.036

[25] Ranogajec I, Jakić-Razumović J, Puzović V, Gabrilovac J. Prognostic value of matrix metalloproteinase-2 (MMP-2), matrix metalloproteinase-9 (MMP-9) and aminopeptidase N/CD13 in breast cancer patients. Med Oncol. 2012;29(2):561–9. 21611838 10.1007/s12032-011-9984-y

[26] Mina-Osorio P. The moonlighting enzyme CD13: old and new functions to target. Trends Mol Med. 2008;14(8):361–71. 18603472 10.1016/j.molmed.2008.06.003 PMC7106361

[27] Lendeckel U, Karimi F, Al Abdulla R, Wolke C. The role of the ectopeptidase APN/CD13 in cancer. Biomedicines. 2023;11(3):724. 36979703 10.3390/biomedicines11030724 PMC10045183

[28] Ahlawat P, Phutela K, Bal A, Singh N, Sharma S. Therapeutic potential of human serum albumin nanoparticles encapsulated actinonin in murine model of lung adenocarcinoma. Drug Deliv. 2022;29(1):2403–13. 35892161 10.1080/10717544.2022.2067600 PMC9336490

[29] Holstein SA, Heckman CA, Davies FE, Morgan GJ, Gelius SS, Lehmann F. Aminopeptidases in cancer, biology and prospects for pharmacological intervention. Curr Cancer Drug Targets. 2022;23(1):25–46. 35747970 10.2174/1568009622666220623112605

[30] Yang J, Shen C, Zhu T, Qian Q, Diao X, Huang W, et al. An aminopeptidase N-based color-convertible fluorescent nano-probe for cancer diagnosis. Biomater Sci. 2023;11(8):2809–17. 36826224 10.1039/d3bm00007a

[31] Deng W, Liu H, Luo S, Clarke J, Glass C, Su L, et al. APOB genotypes and CDH13 haplotypes in the cholesterol-related pathway genes predict non-small cell lung cancer survival. Cancer Epidemiol Biomarkers Prev. 2020;29(6):1204–13. 32238407 10.1158/1055-9965.EPI-19-1262 PMC7269811

[32] Sun L, Zhao H, Ding XY, Yang K, Wang GS, Chen JM, et al. Clinicopathological features of hepatocellular carcinoma with metabolic risk factors. J Hepatocell Carcinoma. 2023;10:833–46. 37304209 10.2147/JHC.S412129 PMC10257051

[33] Lin Z, Ji X, Tian N, Gan Y, Ke L. APOB is a potential prognostic biomarker in hepatocellular carcinoma. Discov Oncol. 2024;15(1):28. 38310202 10.1007/s12672-024-00877-6 PMC10838261

[34] Sawada M, de Fátima Mello Santana M, Reis M, de Assis SIS, Pereira LA, Santos DR, et al. Increased plasma lipids in triple-negative breast cancer and impairment in HDL functionality in advanced stages of tumors. Sci Rep. 2023;13(1):8998. 37268673 10.1038/s41598-023-35764-7 PMC10238519

[35] Ng PY, Nafi SNM, Jalil NAC, Kueh YC, Lee YY, Zin AAM. Immunohistochemical expression of apolipoprotein B and 4-hydroxynonenal proteins in colorectal carcinoma patients: a retrospective study. Croat Med J. 2023;64(1):29–36. 36864816 10.3325/cmj.2023.64.29 PMC10028567

[36] Gong Y, Zhang L, Bie P, Wang H. Roles of ApoB-100 gene polymorphisms and the risks of gallstones and gallbladder cancer: a meta-analysis. PLoS ONE. 2013;8(4): e61456. 23637837 10.1371/journal.pone.0061456 PMC3630192

[37] Kalló G, Bertalan PM, Márton I, Kiss C, Csősz É. Salivary chemical barrier proteins in oral squamous cell carcinoma-alterations in the defense mechanism of the oral cavity. Int J Mol Sci. 2023;24(17):13657. 37686462 10.3390/ijms241713657 PMC10487546

[38] Li W, Lyu W, Liu S, Ruan F, Zhang X. GLP1R boosts survival, migration and invasion of endometrial cancer cells and protects against ferroptotic cell death. J Obstet Gynaecol. 2024;44(1):2301324. 38269495 10.1080/01443615.2023.2301324

[39] Zhang X, Zhang L, Wang B, Zhang X, Gu L, Guo K, et al. GLP-1 receptor agonist liraglutide inhibits the proliferation and migration of thyroid cancer cells. Cell Mol Biol. 2023;69(14):221–5. 38279433 10.14715/cmb/2023.69.14.37

[40] Li YW, Bai L, Dai LX, He X, Zhou XP. Chromosomal and genetic analysis of a human lung adenocarcinoma cell line OM. Chin Med J (Engl). 2016;129(4):405–9. 26879013 10.4103/0366-6999.176066 PMC4800840

[41] Zhang H, Yu Y, Wang J, Han Y, Ren T, Huang Y, et al. Macrophages-derived exosomal lncRNA LIFR-AS1 promotes osteosarcoma cell progression via miR-29a/NFIA axis. Cancer Cell Int. 2021;21(1):192. 33794884 10.1186/s12935-021-01893-0 PMC8017664

[42] Singh J, Narayan G, Dixit VK. The long intergenic non-coding RNA LINC01140 modulates gastric cancer phenotypes and cancer cell lines aggressiveness. Dig Liver Dis. 2024;56(10):1776–83. 38556409 10.1016/j.dld.2024.03.006

[43] Wu N, Chen J, Lin T, Zhong Z, Li M, Yu Y, et al. Identification of AP0024981 and LINC01871 as prognostic biomarkers and therapeutic targets for distant metastasis of colorectal adenocarcinoma. Cancer Med. 2024;13(1): e6823. 38083905 10.1002/cam4.6823 PMC10807603

[44] Yu Y, Dong G, Li Z, Zheng Y, Shi Z, Wang G. circ-LRP6 contributes to osteosarcoma progression by regulating the miR-141-3p/HDAC4/HMGB1 axis. Int J Oncol. 2022;60(4):38. 35211755 10.3892/ijo.2022.5328 PMC8878724

[45] Lin Y, Zhang B, Su L, Wei J, Chen Z, Wu M. Expression of lncRNA DLX6-AS1 in patients with hepatic carcinoma and its prognostic value. Biotechnol Genet Eng Rev. 2024;40(4):3976–87. 37078565 10.1080/02648725.2023.2204706

[46] Liu C, Chen Z. ZC3H13 knockdown enhances the inhibitory effect of sevoflurane on gastric cancer cell malignancy by regulating the N6-methyladenosine modification of the lncRNA DLX6-AS1. Heliyon. 2024;10(16): e35722. 39220970 10.1016/j.heliyon.2024.e35722 PMC11365301

[47] Gupta S, Silveira DA, Mombach JCM, Hashimoto RF. The lncRNA DLX6-AS1/miR-16-5p axis regulates autophagy and apoptosis in non-small cell lung cancer: a Boolean model of cell death. Noncoding RNA Res. 2023;8(4):605–14. 37767112 10.1016/j.ncrna.2023.08.003 PMC10520667

[48] Park SM, Gaur AB, Lengyel E, Peter ME. The miR-200 family determines the epithelial phenotype of cancer cells by targeting the E-cadherin repressors ZEB1 and ZEB2. Genes Dev. 2008;22(7):894–907. 18381893 10.1101/gad.1640608 PMC2279201

[49] Arunkumar G, Deva Magendhra Rao AK, Manikandan M, Prasanna Srinivasa Rao H, Subbiah S, Ilangovan R, et al. Dysregulation of miR-200 family microRNAs and epithelial-mesenchymal transition markers in oral squamous cell carcinoma. Oncol Lett. 2018;15(1):649–57. 29375721 10.3892/ol.2017.7296 PMC5766066

[50] Cao M, Tian K, Sun W, Xu J, Tang Y, Wu S. MicroRNA-141-3p inhibits the progression of oral squamous cell carcinoma via targeting PBX1 through the JAK2/STAT3 pathway. Exp Ther Med. 2022;23(1):97. 34976139 10.3892/etm.2021.11020 PMC8674974

[51] Sannino G, Marchetto A, Kirchner T, Grünewald TGP. Epithelial-to-mesenchymal and mesenchymal-to-epithelial transition in mesenchymal tumors: a paradox in sarcomas? Cancer Res. 2017;77(17):4556–61. 28811330 10.1158/0008-5472.CAN-17-0032

[52] Li J, Chen S, Xiao J, Ji J, Huang C, Shu G. FOXC1 transcriptionally suppresses ABHD5 to inhibit the progression of renal cell carcinoma through AMPK/mTOR pathway. Cell Biol Toxicol. 2024;40(1):62. 39093497 10.1007/s10565-024-09899-w PMC11297099

[53] Kong XP, Yao J, Luo W, Feng FK, Ma JT, Ren YP, et al. The expression and functional role of a FOXC1 related mRNA-lncRNA pair in oral squamous cell carcinoma. Mol Cell Biochem. 2014;394(1–2):177–86. 24889262 10.1007/s11010-014-2093-4 PMC4118037

[54] Che H, Che Y, Zhang Z, Lu Q. Long non-coding RNA LINC01929 accelerates progression of oral squamous cell carcinoma by targeting the miR-137-3p/FOXC1 axis. Front Oncol. 2021;11: 657876. 33968763 10.3389/fonc.2021.657876 PMC8097103

[55] Han JM, Oh KY, Choi SJ, Lee WW, Jin BH, Kim JH, et al. Antitumor activity of afatinib in EGFR T790M-negative human oral cancer therapeutically targets mTOR/Mcl-1 signaling axis. Cell Oncol (Dordr). 2025;48(1):123–38. 38888847 10.1007/s13402-024-00962-6 PMC11850456

[56] Jiang Y, Fang X, Xiang Y, Fang T, Liu J, Lu K. Afatinib for the treatment of NSCLC with uncommon EGFR mutations: a narrative review. Curr Oncol. 2023;30(6):5337–49. 37366888 10.3390/curroncol30060405 PMC10297373

[57] Seiwert TY, Jagadeeswaran R, Faoro L, Janamanchi V, Nallasura V, El Dinali M, et al. The MET receptor tyrosine kinase is a potential novel therapeutic target for head and neck squamous cell carcinoma. Cancer Res. 2009;69(7):3021–31. 19318576 10.1158/0008-5472.CAN-08-2881 PMC2871252

[58] Ota T, Masuda N, Matsui K, Yamada T, Tanaka N, Fujimoto S, et al. Successful desensitization with crizotinib after crizotinib-induced liver injury in ROS1-rearranged lung adenocarcinoma. Intern Med. 2019;58(18):2651–5. 31178493 10.2169/internalmedicine.2554-18 PMC6794186

[59] Chu LP, Franck D, Parachoniak CA, Gregg JP, Moore MG, Farwell DG, et al. MET genomic alterations in head and neck squamous cell carcinoma (HNSCC): rapid response to crizotinib in a patient with HNSCC with a novel MET R1004G mutation. Oncologist. 2019;24(10):1305–8. 31391294 10.1634/theoncologist.2019-0230 PMC6795166

[60] Polosukhina D, Singh K, Asim M, Barry DP, Allaman MM, Hardbower DM, et al. CCL11 exacerbates colitis and inflammation-associated colon tumorigenesis. Oncogene. 2021;40(47):6540–6. 34625710 10.1038/s41388-021-02046-3 PMC8629429

[61] Payne AS, Cornelius LA. The role of chemokines in melanoma tumor growth and metastasis. J Invest Dermatol. 2002;118(6):915–22. 12060384 10.1046/j.1523-1747.2002.01725.x

[62] Ding Q, Lu P, Xia Y, Ding S, Fan Y, Li X, et al. CXCL9: evidence and contradictions for its role in tumor progression. Cancer Med. 2016;5(11):3246–59. 27726306 10.1002/cam4.934 PMC5119981

