# Supplementary material for: Identification of novel biomarkers involved in oral squamous cell carcinoma by whole transcriptome sequencing and bioinformatics analysis
Source: Cancer Cell Int. 2025 Jul 22;25:277. doi: 10.1186/s12935-025-03913-9 (PMC12285194; doi:10.1186/s12935-025-03913-9)
Supplement: Supplementary file 1 — Additional file 1. [file 12935_2025_3913_MOESM1_ESM.doc]

**Supplementary Table1** Clinical Pathology Data Sheet

| Patient Number | Age  (years) | gender | Location | TNM staging  (8th edition) | Pathological type |
| --- | --- | --- | --- | --- | --- |
| 1 | 58 | Female | Buccal mucosa | T3N1M0 | Squamous cell carcinoma |
| 2 | 49 | Male | Buccal mucosa | T2N2M0 | Squamous cell carcinoma |
| 3 | 44 | Female | Tongue | T3N1M0 | Squamous cell carcinoma |
| 4 | 52 | Male | Tongue | T3N2M0 | Squamous cell carcinoma |
| 5 | 59 | Female | Buccal mucosa | T2N1M0 | Squamous cell carcinoma |
